# Effects of Spinal Decompression and Segmental Spinal Instrumentation on Lower Limb Functionality in Patients with Spinal Osteoarthritis

**DOI:** 10.3390/life14091072

**Published:** 2024-08-27

**Authors:** Milan Mirković, Filip Kukić, Dragan Mirkov, Dejan Marinković, Lazar Mičeta, Sanja Mirković, Biljana Božić Nedeljković, Zoran Baščarević

**Affiliations:** 1 Institute for Orthopedic Surgery “Banjica”, 11000 Belgrade, Serbia; drckmilan@yahoo.com (M.M.); lazar.miceta@iohbb.edu.rs (L.M.); zoran.bascarevic@iohbb.edu.rs (Z.B.); 2 Faculty of Physical Education and Sports, University of Banja Luka, 78000 Banja Luka, Bosnia and Herzegovina; 3 Faculty of Sport and Physical Education, University of Belgrade, 11000 Belgrade, Serbia; dragan.mirkov@fsfv.onmicrosoft.com (D.M.);; 4 Institute of Physiology and Biochemistry “Ivan Djaja”, Faculty of Biology, University of Belgrade, 11000 Belgrade, Serbia; biljana@bio.bg.ac.rs

**Keywords:** quality of life, healthcare, biomechanics, spine mechanics, gate

## Abstract

Spinal osteoarthritis, a degenerative condition of the spine, significantly impairs quality of life, causing pain, stiffness, and limited mobility. Treatment primarily aims to improve functionality and quality of life. This study investigated the effects of lower back surgery (e.g., spinal decompression with vertebra stabilization) on upper-limb maximum strength, lower limb functionality, and quality of life in osteoarthritis patients. A total of 16 patients (♀ = 10 and ♂ = 6) patients from the orthopedic clinic who were diagnosed with osteoarthritis based on MRI and clinical symptoms underwent the surgery. Their handgrip strength, lower limb functionality (6 min walk test), and quality of life (Flanagan quality of life scale) were assessed before and after the surgery. A repeated measures ANOVA was conducted to assess differences in these metrics pre- and post-surgery across the entire patient cohort, as well as within gender-specific subgroups. A large positive effect was seen in the 6 min walk (*p* = 0.02, *d* = −0.83) test but not in the handgrip test or quality of life. However, female patients showed a trend towards better quality of life. The results of this study provide evidence for the effectiveness of surgery in the treatment of spinal osteoarthritis and underscore the need for personalized treatment approaches in the treatment of spinal osteoarthritis.

## 1. Introduction

Spinal osteoarthritis (sOA), a degenerative joint disease affecting the spine, poses significant challenges to patients’ quality of life [1] and often leads to pain [2], stiffness, and limited mobility [1,3]. Both peripheral and central symptoms of OA may reflect in a variety of symptoms [4,5]. The most common symptoms of sOA are lower back pain (e.g., dull localized pain or radiating pain), stiffness (e.g., morning stiffness and inactivity-related stiffness), limited range of motion (e.g., difficulty in bending and twisting caused by joint degeneration), muscle weakness, numbness or tingling, sciatica, and instability [1,3,6,7]. All these symptoms impair one’s functionality and quality of life, leading them to seek treatments that remove symptoms. In cases where conservative treatments fail to alleviate symptoms, surgical procedures such as spinal decompression and segmental spinal instrumentation are often performed to address structural abnormalities and alleviate symptoms as they appear to be more effective than nonsurgical treatments [8]. Customized interventions targeting the complexity of sOA can improve accessibility and effectiveness following specialized surgery, thereby complementing the comprehensive treatment of those affected. This approach includes a multidisciplinary framework, involving various healthcare professionals, as well as education that emphasizes the importance of physical activity and the integration of technology-enabled rehabilitation methods.

While the primary focus of surgery in sOA patients is often on improving lumbar spine function and relieving associated pain [8,9], the potential impact of such interventions on other aspects of physical function, particularly upper-limb strength and lower limb functionality, is less well understood. Understanding these effects is crucial for comprehensive patient care and optimization of postoperative rehabilitation strategies. To achieve this, functional assessments should be performed to measure upper-limb strength and lower limb functionality using validated outcome measures.

Considering this, handgrip strength testing has been shown to be an important surrogate for predicting of outcomes in aging adults [10] and postoperative changes in spine–pelvic alignment [11]. McGrath et al. [12] recommended using this test as an overarching assessment that provides information on strength capacity and general health. Understandably, surgery on the lower back may not affect the upper limbs. However, checking overall strength capacity before and after the surgery is an important marker of patients’ preparedness for and recovery from the surgery. The 6 min walk test, on the other hand, is an excellent indicator of lower limb functionality as well as overall functionality, depending on the specificity of the subjects performing it [13]. Most patients can perform it quickly and confidently, which may be the reason why it is frequently used in clinical practice and research studies as an objective measure of functional status in patients with moderate-to-severe impairment [14].

The aim of this study was to investigate the effects of lower back surgery (e.g., spinal decompression and segmental spinal instrumentation) on upper-limb maximum strength, lower limb functionality, and quality of life in osteoarthritis patients. We hypothesized that upper-limb maximum strength would not be affected by surgery, while gait functionality and quality of would improve.

## 2. Materials and Methods

### 2.1. Subjects

This study included 16 (♀ = 10 and ♂ = 6) patients from the orthopedic clinic who were diagnosed with osteoarthritis using MRI and clinical symptoms. Furthermore, the subjects were graded using the adequate scale according to the Kellgren–Lawrence (KL) classification which provides a quantitative score and a qualitative explanation of the degree of osteoarthritis [15]. Two patients scored 2 (12.5%) on the KL scale, nine scored 3 (56.25%), and five scored 4 (31.25%). All female patients scored 3, while two male patients scored 2, three scored 3, and five scored 4. The main age of the sample was 55.9 ± 10.5 years. The mean period between the surgery and the post-test was 22.7 weeks (min = 9 weeks, max = 44 weeks) (see Supplementary Table S1). The inclusion criteria were clinical symptoms of lower back and lower limb pain, and impaired gait, along with radiological signs of spinal stenosis and degenerative spinal changes (e.g., ossification, degenerative disk disease) observed on RTG, MRI, and CT. The exclusion criteria were the diagnosis of any neural condition (e.g., cerebral paralysis), the ability to walk longer distances without pain or any other condition, and the ability to function after surgery despite treatment. All participants had previously undergone unsuccessful treatment with medication and physical therapy. All participants were informed of the aim of the study and only those who signed their informed consent were included in the analysis. This study was approved by the ethical committee of the Institute for Orthopedic Surgery “Banjica” No I-264/1. It was conducted following the Helsinki Declaration for human subjects [16].

### 2.2. Surgical Procedure

All participants underwent the same surgery: spinal decompression and segmental spinal instrumentation. This surgery consists of placing screws transpediculary into the lumbal and sacral vertebrae (L1–S1), followed by decompression. The decompression was performed by removing bone and soft tissue parts that created pressure and narrowed the spinal canal. Finally, the screws were connected with rods that stabilized the spine. After 24 h post surgery, the drain was removed, and patients started mobilizing.

### 2.3. Physical Fitness Measures

The handgrip test was performed in a seated position using a JAMAR plus handgrip dynamometer. Subjects were instructed to squeeze the dynamometer as hard as possible for three seconds [11]. The test was repeated three times with a between-trial rest of one minute, and the strongest trial was recorded in kilograms.

### 2.4. Lower-Limb Functionality

A 6 min walk test was used to assess subjects’ lower limb functionality prior to and post surgical treatment following the procedure proposed by the American Thoracic Society [17]. This test is a commonly used assessment to measure functional capacity in various populations [18]. Subjects were instructed to walk as far as possible in 6 min on a flat, hard surface, with encouragement to pace themselves and take breaks if needed. A straight, 30 m corridor or track was marked, and the distance covered was recorded at the end of the test. Observations of symptoms or difficulties experienced during the test were noted. The recorded distance provides valuable insight into the participant’s functional capacity and can be used to assess changes over time, making the 6 min walk test a practical and reliable tool for clinical and research purposes [19].

### 2.5. Quality of Life

The Flanagan quality of life scale [20] was used to assess patients’ quality of life prior and after the surgery. It consists of 15 items spanning five domains: physical and material well-being; relationships with others; social, community, and civic activities; personal development and fulfillment; and recreation. Each item assesses both the individual’s satisfaction and the importance of that item in their quality of life [21]. This scale has been shown to be a valid and reliable instrument for measuring quality of life across patient groups [20,22].

### 2.6. Statistical Analysis

The statistical procedures were performed with the statistical software JASP (v 0.18.1, University of Amsterdam, The Netherlands). Descriptive statistics were displayed for mean, standard deviation, minimum, and maximum. Correlation analysis was used to evaluate the association of the duration between the surgery and post-test with outcome measures. A repeated measures ANOVA was used to examine the difference in handgrip strength and 6 min walk test score before and after the surgery, and the interactions with gender and KL score were also tested. The significance was set at *p* < 0.05. The effect sizes were calculated and interpreted according to Cohen as small = 0.2–0.5, moderate = 0.5–0.8, large = 0.8–1.2, and very large > 1.2 [23]. Given that we used a sample of convenience, G*Power analysis was employed to determine the minimum effect size for the given sample size, resulting in an effect size of 0.45 for the lower bound for a small effect size.

## 3. Results

Pre- and post-treatment descriptive statistics for handgrip, 6 min walk test, and quality of life are shown in Table 1. There was no significant correlation (r > 0.05) of the time past between the surgery and post-treatment tests.

The repeated measures ANOVA showed that the treatment had a large positive effect on the 6 min walk test but not on the handgrip test (Table 2).

**Figure 1:**
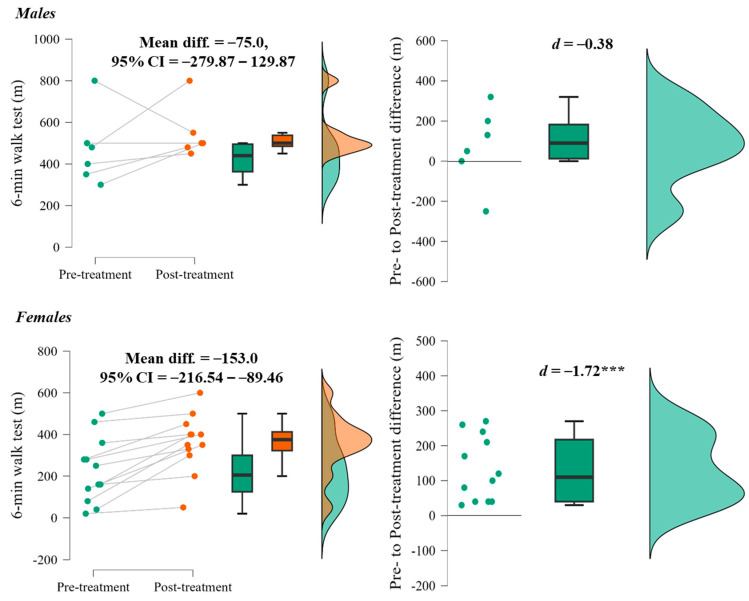
Differences in 6 min walk test in males and females. Note: *** Significant at *p* < 0.001.

There was no significant interaction between the factors of surgery and gender (F = 0.765, *p* = 0.395). However, when tested independently and checked for each gender, the effects were not significant in male patients (*p* = 0.39) but significant (*p* < 0.001) and greater in female patients (Figure 1). Two male patients had worse outcomes at post-treatment recovery, while all female patients performed better after treatment.

**Figure 2:**
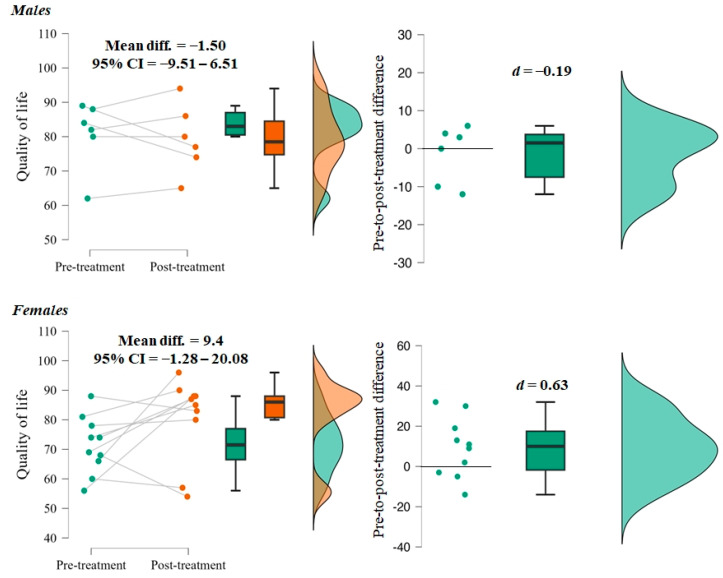
Differences in quality of life in males and females.

There was no significant difference in reported quality of life pre- and post surgery (F = 0.683, *p* = 0.422). However, female patients showed a trend towards a better quality of life (F = 1.99, *p* = 0.078), while no changes were observed in males (F = −0.481, *p* = 0.651) (Figure 2). It is also notable that the effect size was moderate in females and trivial in males, and that the majority of female patients showed improvements in their quality of life (Figure 2).

**Figure 3:**
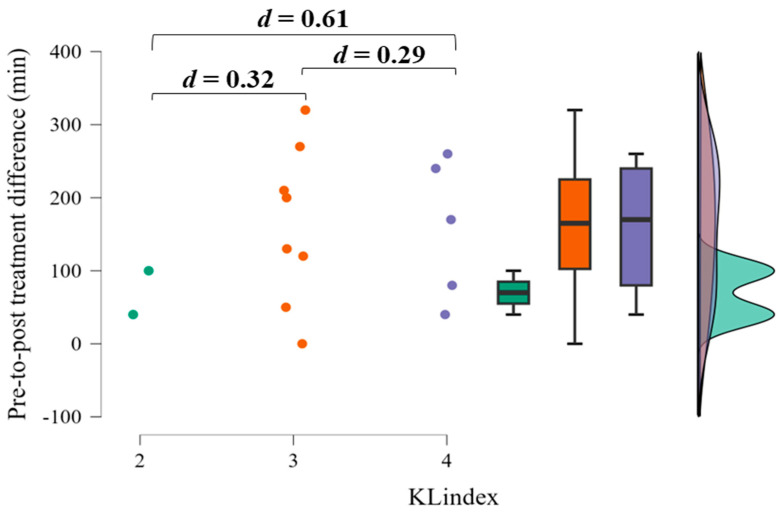
Effect size analysis of the differences related to osteoarthrosis level.

The KL score had no significant effect (F = 0.289, *p* = 0.754) on the differences before and after surgery in the 6 min walk test. However, it is noteworthy that the Bonferroni post hoc test (Table 3) and the effect size analysis (Figure 3) showed a trend towards better treatment effects in the 6 min walk test in patients with a KL index of 3 and 4.

## 4. Discussion

The aim of the present study was to investigate the efficacy of spinal decompression and segmental spinal instrumentation surgery on upper-limb strength, lower limb functionality, and quality of life in sOA patients. As expected, no statistically significant improvement in handgrip strength was observed after surgery, while a significant improvement in lower limb functionality was observed. Considering quality of life, we partially confirmed our hypothesis as no statistically significant improvement occurred, but there was a trend among female patients suggesting improvement. These results indicate the potential of surgical interventions to restore lower limb functionality. This was noticeably pronounced in sOA patients with a KL score of 4 when compared with those with a score of 2.

A gender-specific analysis revealed a different response to the surgical interventions, with females showing a more marked improvement in lower limb functionality compared to males. Of note, all female patients showed improved functionality after surgery. Conversely, the effects were less pronounced in the male patients, with one of them showing a negative outcome and one showing a similar result in the 6 min walk test after surgery as before. Noteworthy is the case of a male patient whose functionality deteriorated after surgery, although his pre-surgery performance was very high (the best of all subjects). It could therefore be the case that this patient was very efficient before surgery and that his performance decreased after the surgery due to the reduced physical activity. In addition, a significant proportion of male and female patients were able to walk more than 200 m after surgery. This underlines the potential for improved mobility after surgery, which is in line with previous research that showed improvements in physical performance, including walking and balancing, after decompression lumbar spinal surgery [24]. Gender- and patient-specific differences from our study underscore the need for personalized treatment approaches in the treatment of sOA.

While the overall quality of life remained constant, when examined by gender, female patients were more likely to report an improvement in a gender-specific analysis. Of the male patients, only three reported a higher quality of life, compared with seven of the female patients in our sample. A study investigating surgical versus nonsurgical therapy for lumbar spinal stenosis showed significant improvements in quality of life measured by a 36-item short-form general health survey [8]. Furthermore, a large meta-analysis of studies investigating quality of life in patients undergoing spine surgery highlighted that surgery was associated with improved quality of life [25]. The authors also pointed out that baseline quality of life varied among patients, which could influence surgical outcomes. This could partially explain the variation in quality of life in our study. Analysis of the relationship between initial diagnosis severity, as indicated by KL scores, and surgical outcomes revealed a trend in which patients with higher KL scores tended to have more favorable postoperative outcomes. This finding is of clinical significance as it suggests that patients with a more severe baseline condition may benefit more from surgery. However, further research is needed to fully clarify the nuanced interplay between baseline severity and surgical efficacy.

### Limitations

The lack of a control group and the relatively small sample size were the main limitations of this study. Further limitations include limited age range, relatively long span between the surgery and post-surgery assessment. All these may have restricted the granularity of data on post-surgery recovery across various age groups. Nonetheless, to our knowledge, this is the first study to examine the effects of sOA surgery on patient functionality, offering significant insights based on gender differences.

## 5. Conclusions

The results of this study contribute to the growing body of evidence for the effectiveness of surgery in the treatment of sOAt. The observed gender differences and the influence of the severity of the initial diagnosis underline the importance of individualized treatment strategies in optimizing patient outcomes. Further research efforts are imperative to validate and refine these findings to ultimately improve clinical decision-making and improve patient care in the field of sOA management.

## Figures and Tables

**Table 1:** Descriptive statistics.

Variable	Pre-Treatment				Post-Treatment			
	Mean	Std. Deviation	Min	Max	Mean	Std. Deviation	Min	Max
Handgrip (kg)	29.17	11.52	12	58	28.69	9.80	4	48
6 min walk (m)	273.68	184.02	20	800	428.75	145.64	50	800
Quality of life	74.94	10.51	56	89	80.25	12.31	54	96

**Table 2:** Differences in handgrip strength and 6 min walk test on general level.

Variables	Mean Difference	95% Conf. Int.		*p*	Effect Size	95% Conf. Int.	
		Lower	Upper			Lower	Upper
6 min walk test (m)	−113.89	−189.69	−38.31	0.002	−0.83	−1.394	−0.307
Handgrip strength (kg)	0.78	−2.44	4.00	0.617	0.12	−0.345	0.582

**Table 3:** Differences in pre- and post-treatment 6 min walk test performance related to initial KL score.

KL Score	Mean Difference		95% CI for Mean Difference		SE	t
			Lower	Upper		
2	3	−46.67	−345.39	252.06	113.13	−0.41
	4	−88.00	−407.71	231.71	121.08	−0.73
3	4	−41.33	−254.48	171.81	80.72	−0.51

Note: KL score—Kellgren–Lawrence score.

## Data Availability

Data are available upon reasonable request to drckmilan@yahoo.com.

## Supplementary Materials

The following supporting information can be downloaded at: https://www.mdpi.com/article/10.3390/life14091072/s1, Table S1: Descriptive statistics. Reference [26] is cited in Supplementary Materials.
