# Supplementary material for: Effects of Spinal Decompression and Segmental Spinal Instrumentation on Lower Limb Functionality in Patients with Spinal Osteoarthritis
Source: Life (Basel). 2024 Aug 27;14(9):1072. doi: 10.3390/life14091072 (PMC11433251; doi:10.3390/life14091072)
Supplement: Supplementary file 1 [file life-14-01072-s001.zip › life-3146517-supplementary.pdf]

Supplement Table S1. Descriptive statistics.

Variable	Sex	Median	Mean	Std. Deviation	Minimum	Maximum
Age (yrs)	Male	58.5	58.1	9.5	42.3	70.6
	Female	55.5	54.5	11.7	34	71.8
Below 40 yrs		Male = 0, Female = 1, ∑ = 1				
40-50 yrs		Male = 1, Female = 3, ∑ = 4				
50-60yrs		Male = 2, Female = 2, ∑ = 4				
60 and more yrs		Male = 3, Female = 4, ∑ = 7				
Pre-to-Post test period (Weeks)	Male	14	17.6	7.8	12	31
	Female	22	23.6	10.6	9	44
BMI	Male	29.6	31	6.1	25.8	42.5
	Female	27.7	26	9.4	2.2	37.3
BMI classification	Male	Not-obese = 4, Obese = 2				
	Female	Not-obese = 7, Obese = 3				

Note. BMI classification according to WHO[1]. Overweight category was not used due to small sample size. ∑ - males and females summed together.

1. A Healthy Lifestyle - WHO Recommendations Available online: https://www.who.int/europe/news-room/fact-sheets/item/a-healthy-lifestyle---who-recommendations (accessed on 15 August 2024).
